
JOURNAL INFORMATION
==============================
NLM Title Abbreviation: ScientificWorldJournal
Journal ID: ScientificWorldJournal
NLM Journal ID: 101131163
Journal ID: TSWJ

The Scientific World Journal

ISSN: 2356-6140
EISSN: 1537-744X
Publisher: Wiley

ARTICLE INFORMATION
==============================
PMCID: PMC6084053
DOI: 10.1100/tsw.2001.446
Article ID: 531578
Article version: 1
Subjects: Extended Abstract

Blood Pressure and Cardiovascular Tone: Role of CGRP Family of Peptides

Wimalawansa Sunil J.
Chief of Endocrinology and Metabolism, Robert Wood Johnson Medical School, New Brunswick, NJ, USA
Publication date: 2001
Electronic publication date: 2001 May 1
Volume: 1
First page: 32
Last page: 32
Copyright: Copyright © 2001 Sunil J. Wimalawansa.
Copyright year: 2001
License: This is an open access article distributed under the Creative Commons Attribution License, which permits unrestricted use, distribution, and reproduction in any medium, provided the original work is properly cited.
License URL: https://creativecommons.org/licenses/by/3.0/

==============================
4th International Meeting on Calcitonin Gene-Related Peptide (CGRP)
*TheScientificWorld* (2001) 1(S1), 32
ISSN 1532-2246; DOI 10.1100/tsw.2001.446

### BLOOD PRESSURE AND CARDIOVASCULAR TONE: ROLE OF CGRP FAMILY OF PEPTIDES

Sunil J. Wimalawansa, M.D., Ph.D.
Chief of Endocrinology and Metabolism, Robert Wood Johnson Medical School, New Brunswick, NJ

Calcitonin gene-related peptide (CGRP) belongs to a family of peptides (adrenomedullin, amylin and calcitonin), most of which have significant effects on the cardiovascular system[1]. CGRP is the most potent endogenous vasodilatory peptide and is the most studied of the peptides in this family[2]. CGRP and its receptors are widely distributed throughout the vascular system. Localization of i-CGRP and its receptors in the heart, correlates well with the postulated functions of this peptide (i.e., increase in heart rate, atrial contractility, and increase in coronary blood flow). CGRP and ADM have direct effects on capacitance blood vessels, and administration leads to peripheral vasodilation and reduced vascular resistance. Injection or an infusion of physiological doses of CGRP and ADM has both significant effects on the blood pressure; cardiac performance, and blood supply to critical organs. Effects of CGRP (and its antagonists) have been examined as potential therapeutic agents in controlling hypertension, cardiac failure, cardiac arrhythmia, migraine headaches, Renaud’s syndrome, preeclampsia, etc. Animal studies have revealed that in the presence of progesterone, administration of CGRP can alleviate both premature delivery and preeclampsia-like syndrome. The role of CGRP as a potential therapeutic agent and its mechanism of actions in alleviating pregnancy-induced hypertension will be discussed in detail[3].

### REFERENCES

1. Wimalawansa, S.J. (1997) Amylin, calcitonin gene-related peptide, calcitonin, and adrenomedullin: A peptide super family. Crit. Rev. Neurobiol. 11, 167–239.
2. Wimalawansa, S.J. (1996) Calcitonin gene-related peptide and its receptors: molecular genetics, physiology, pathophysiology, and therapeutic potentials. Endocrine Rev. 17, 533–585.
3. Yallampalli, C. and Wimalawansa, S.J. (1998) Calcitonin gene-related peptide is a mediator of vascular adaptation during hypertension in pregnancy. Trends Endocrinol Metab. 9, 113–117.
